# New Members

**Published:** 1890-05

**Authors:** 


NEW MEMBERS.

Alexander, W. P., Cleburne; Akard, G. W., Springtown; Bond, Geo. D., Hillsboro; Berg, L. M., Laredo; Burch, J. D., Aurora; Bundy, Z. T., Midlothian; Brown, W. E., Gatesville; Cooper, S. C., Jefferson; Davis, J. A., Austin; Embry, J. A., Decatur; Fry, J. M., Elmo; Gale, Robert, Dallas; Gracey, J. A., Waxahachie; Gallaher, J. W., Graham; Holloway, J. A., Round Rock; Kimmins, R. S., Iredell; Kemp, Jos., Walnut; Karnes, T. C., West Point; Kuykendall, A. R., Kopperl; Kirkpatrick, D. F., Denton; Lee, B. F. Rockdale; McLane, W. S., Itaska; McDaniel, J. H., Centreville; Mayes, C. M., Archer; Moody, R. E., Sweetwater; O'Barr, J. T., Ledbetter; Orr, C. S., Bethel; Oates, T. F., Mexia; Prince, J. E., Big Springs; Reuss, J. H., Cuero; Riley, H., Bowie; Raines, C. B., Jr., Mineral Wells; Randall, L. J., Aurora; Rawlins, B. L., Dallas; Strain, W. T. McCoy; Sparkmen, J. T., Alvord; Stevens. H. C., Arlington; Smith, J. R., Hillsboro; Snyder, E. W., Benbroke; Thomason, B. R., Era; Towns, J. M., Joshua; Tye, R. P., Clarendon; Talbot, R. D., Birdville; Worsham, B. M., Waxahachie; Walkins, W. B. W., Eureka; Weathered, R. J., Osceola; Welch, W. C., Commerce; Young, F. E., Brownwood. Total, 48.

